# Alleviating Chilling Injury in Stored Pomegranate Using a Single Intermittent Warming Cycle: Fatty Acid and Polyamine Modifications

**DOI:** 10.1155/2021/2931353

**Published:** 2021-11-11

**Authors:** Leila Taghipour, Majid Rahemi, Pedram Assar, Asghar Ramezanian, Seyed Hossein Mirdehghan

**Affiliations:** ^1^Department of Horticultural Science, School of Agriculture, Shiraz University, P.O. Box: 71441-65186, Shiraz, Iran; ^2^Department of Horticultural Science, College of Agriculture, Jahrom University, P.O. Box: 74135-111, Jahrom, Iran; ^3^Department of Horticultural Science, College of Agriculture, Vali-Asr University of Rafsanjan, P.O. Box: 518, Kerman, Iran

## Abstract

Pomegranate is a perishable superfruit with important human health-promoting phytochemicals. The use of cold storage is inevitable for its long-term preservation. As pomegranate is sensitive to temperatures below 5°C, it is therefore necessary and worthwhile to introduce a postharvest technique that is safe, applicable, and commercially acceptable to maintain the fruit quality under a cold storage condition. The efficacy of intermittent warming (IW) in the form of a single warming period (1 day at 20°C with 70% relative humidity (RH) before returning the treated fruit to storage) during the cold storage of ‘Rabab-e-Neyriz' pomegranate (70 days at 2 ± 0.5°C and 90 ± 5% RH) was evaluated. To find the best treatment time, warming was performed at 4 temporary interruption points in storage (after 15, 25, 35, or 45 days of storage). For each interruption date, the treated fruit were compared to the controls twice, once immediately after treatment and once at the end of the storage period. It was founded that a single warming period at the right time during cold storage (before irreversible damage occurs) activated multiple mechanisms and physiological responses in pomegranate fruit peel that are significantly responsible for alleviating the severity of chilling damage to this commodity. In other words, warming on the 15th day was the most efficient treatment, resulting in better preservation of unsaturated fatty acids from peroxidation, lower malondialdehyde (MDA) production, and preservation of the unsaturated/saturated fatty acids (UFAs/SFAs) ratio (membrane integrity index) in the peel during storage and lower chilling injury symptoms. Moreover, the content of spermine (Spm) and putrescine (Put) (as important antioxidants acting as membrane safety agents) was significantly increased immediately after treatment, followed by a continuous increase in Spm and a higher level of Put compared to control until the end of storage.

## 1. Introduction

Nowadays, there is a worldwide and increasing notion that superfruits and their ingredients and extracts may have the ability to prevent diseases and/or be used as a cure for ailments. Pomegranate fruit (*Punica granatum* L.), known in many countries as the fruit of Eden [1], is a superfruit with excellent taste and great health benefits. Nearly 124 different phytochemicals can be found in pomegranate fruit; interestingly, not limited to the edible part of the fruit, which is likely to mediate in protective mechanisms against a wide range of oxidative and inflammatory human disorders, including cancer, type 2 diabetes, atherosclerosis and cardiovascular diseases [2].

Low-temperature storage is considered the most efficient way to preserve the postharvest quality of horticultural crops for an extended period of time. However, for some tropical or subtropical perishable products, such as pomegranate fruit, too long cold storage could result in a sequence of physiological disorders collectively known as chilling injury (CI), leading to significant loss of quality [3]. Therefore, finding safe, effective, and preferably non-chemical treatments to reduce postharvest losses during cold storage of pomegranate is both worthwhile and inevitable.

Intermittent warming (IW) is a potential environmentally friendly postharvest technique to alleviate chilling injury (CI) in cold-stored products and refers to the periodic exposure of fruit to warm temperatures at 20–27°C during storage [3–7]. The timing of treatment is critical [8], i.e., the initial interruption of cold storage must occur before the chilling damages become irreversible [5, 9]. In addition, it is essential to understand the ideal temperature, duration, and frequency that could be specific to each product and cultivar [9]. There are several hypotheses regarding IW's mechanism of action in alleviating chilling damage, e.g., an induced change in unsaturated fatty acids (UFAs) concentration which is considered involved in membrane safety at low temperatures. It was assumed that shifting the temperature from low to high and then from high to low would probably result in an increment of saturated fatty acids (SFAs) followed by desaturation leading to accumulation of UFAs and a greater degree of unsaturation. This change was suggested to affect membrane fluidity and result in increased tolerance to low temperatures [6, 7, 10]. In addition, it has been reported that IW promotes polyamines (PAs) production in treated tissues [11]. It has been proposed that the mechanism of action for the alleviation of CI by exogenous PAs or treatments that enhance their endogenous values may be related to their ability to bind membranes and to have antioxidant activity [12] which mitigates changes in membrane fluidity and solute leakage [13] and results in membrane stability and delayed disintegration [14].

In contrast to achieving consensus on IW as a potential postharvest method, related research efforts in the new millennium have decreased. It is so important to keep in mind that there are challenges in choosing IW for commercial applications. It means that repeatedly increasing and decreasing the storage temperature is a slow and energy-intensive process. In addition, as an alternative, shifting the product from cold to warm rooms for several times is labor-intensive and requires the accessibility of specific spaces [3]. On the one hand, it is recommended that IW for crops with short shelf life, such as cucumbers, sweet peppers, and zucchini squash, be used more frequently [7, 11, 15]. On the other hand, if the warming is applied too frequently or for too long, an increased loss of quality may occur [16]. The authors believe it could be beneficial, more applicable, and commercially acceptable if only one cycle of IW is adequate to alleviate the incidence of CI and should be investigated for commercially important cultivars. In the other part of our research on the cold storage of ‘Rabab-e-Neyriz' pomegranate fruit, the beneficial effect of one cycle of IW was revealed and related to the promotion of enzymatic and non-enzymatic antioxidant responses [17]. However, as stated by Biswas et al. [3], more research is needed to investigate the mechanisms by which IW alleviates CI, which can lead to alternative novel methods with similar advantages. To the best of our knowledge, more studies are required to evaluate the effects of IW on modifications in membrane fatty acids (FAs) and its fluidity and stability in cold-stored sensitive crops such as pomegranate fruit. Moreover, there is no literature available on the possible beneficial effect of IW via modification of endogenous PAs in pomegranate.

Pomegranate cv. ‘Rabab-e-Neyriz' is a late ripening, exportable and commercial Iranian cultivar. The aim of the present research was to investigate the effects of a single warming period on modifications in FAs and PAs in fruit peel and its relationship with the mitigation of chilling damage to this product.

## 2. Materials and Methods

### 2.1. Plant Material, Experimental Design, and Treatments

Fully mature pomegranates cv. ‘Rabab-e-Neyriz' were picked from a commercial orchard in Neyriz (Fars province, Iran). On the day of collection, the fruit were placed in vented plastic crates and transported to the laboratory in a cold room set to 5°C. Upon arrival at the laboratory, fruit with defects were discarded and the remaining were stored at 2 ± 0.5°C (chilling temperature) and 90 ± 5% RH for 70 days. IW was performed by exposure of the fruit to only one period of high temperature (1 day at 20°C with 70% RH) by shifting the fruit to a warm room during the storage period. To find the best treatment time, 4 temporary interruption points were dedicated to warming, i.e., 15th, 25th, 35th, or 45th days of storage.

The experimental design was factorial based on a complete randomized design with three replications. It included 4 temporal points of interruption in storage (15th, 25th, 35th, or 45th days of storage) ×2 levels of warming regime (warming and control) ×2 levels of sampling time (immediately after treatment and at the end of storage). In other words, there was 4 distinct groups of fruit for each interruption day and 4 abbreviations were assigned: WI, WE, NI, and NE. The first letter explains the warming regime; whether the fruit were warmed (W) or not (N), and the second letter shows the sampling time; whether the samples were taken immediately after the warming regime (I) or at the end of the storage period (E). These groups included: Group A: fruit were removed from storage and immediately warmed by shifting to the warm room, and then sampled without delay (abbreviated to WI; as 15WI, 25WI, 35WI and 45WI). Group B: fruit immediately sampled after removal from storage without warming treatment (abbreviated to NI; as 15NI, 25NI, 35NI, and 45NI). Group C: fruit were removed from storage and immediately shifted to the warm room for warming treatment, then were returned to cold storage and kept until the end of the storage period and sampled at the end (abbreviated to WE; as 15WE, 25WE, 35WE and 45WE). Group D: non-treated fruit without removal from storage, which were sampled at the end of cold storage (was abbreviated to NE; as 15NE, 25NE, 35NE, and 45NE). In other words, for each interruption date, groups B and D served as control for groups A and C, respectively. From this point of view, for each interruption date, the treated fruit were compared with the controls twice, immediately after treatment or at the end of the storage period. The content and composition of the FAs and the amount of PAs and malondialdehyde (MDA) in the peel samples were analyzed. In addition, chilling injury (CI) index in different time-treated fruit was evaluated at the end of the storage followed by a shelf life of 3 days and compared with control. Intact fruit were used for this purpose.

For the preparation of the sample, each husk was carefully cut in the equatorial zone and the peels were manually separated. Peel tissues from 10 fruit in each replicate were combined, frozen in liquid N_2_, and stored at −80°C for later analytical determinations.

### 2.2. MDA Content and CI Index

The level of lipid peroxidation in the peel tissue was measured in terms of MDA content (a product of lipid peroxidation) determined by the thiobarbituric acid (TBA) reaction with minor modification of the method of Heath and Packer [18]. Briefly, 0.25 g of peel sample was homogenized in 5 mL of 0.1% trichloroacetic acid (TCA). The homogenated sample was centrifuged at 10000×g for 5 min. To 250 *μ*L aliquot of the supernatant, 1 mL 20% TCA containing 0.5% TBA was added. The mixture was heated at 95°C for 30 min and then quickly cooled in an ice bath. After being centrifuged at 10000×*g* for 10 min, the absorbance of the supernatant was read at 532 nm and the value for the nonspecific absorption at 600 nm was subtracted. The concentration of MDA was calculated using its extinction coefficient (*ε*) of 155 mM^−1^ cm^−1^ reported as *μ*mol per g of peel fresh weight.

The CI index was assessed separately in each fruit with a 4-point hedonic scale based on the proportion of the peel surface impacted by CI symptoms (dehydration, browning, and pitting) [19]: 0 (no symptoms), 1 (1–25% of damaged area), 2 (26–50% of damaged area) and 3 (>50% of damaged area). The results were expressed as the mean±SD of CI calculated using the following formula:

(1) $$\begin{array}{c} \text{CI}=\sum \left(\text{value of hedonic scale}\times \text{number of fruit with the corresponding scale number}\right)/\left(4\times \text{total number of fruit in the sample}\right). \end{array}$$

### 2.3. Fatty Acids (FAs) Quantification

Total lipids were extracted according to the method of Rui et al. [20]. A gas chromatograph (GC, HP-model 6890) equipped with a flame ionization detector was used to separate and quantify fatty acids according to Mirdehghan et al. [21]. At first, two g of skin was homogenised in 10 mL of chloroform:methanol:0.1 N HCl (200 : 100 : 1). Then, 10 mL of 0.1 N HCl was added before centrifugation at 4000 × *g* for 10 min. It was allowed to the organic phase to be dried. By adding 1 mL of boron trifluoride/methanol at boiling temperature for 10 min, fatty acid methylation was done. Using hexane, methylated fatty acids were extracted and then allowed to be dried and redissolved in 200 *μ*L chloroform before injection. For fatty acid separation and quantification, a HP-Innowax polyethylene glycol capillary column (30 m ×250 *μ*m ×25 *μ*m) and a gradient of temperature (initially 120°C for 2 min and then a rate at 4°C/min to 190°C which was held for 5 min, and final rate at 4°C/min to 242°C) were used. Fatty acids were identified and quantified by comparing retention times and peak areas with authentic standards (Sigma–Aldrich, USA). Results were expressed as mg 100 g^−1^ fresh weight.

### 2.4. Polyamines (PAs) Quantification

Sample preparation and HPLC analysis of PAs was performed according to Mirdehghan et al. [21] with some modifications. For each replicate, 1 g of fresh tissue was homogenized with 10 mL of 5% cold perchloric acid. The homogenate was then centrifuged for 30 min at 20000 × *g*. The resulted supernatant was filtered through a 0.45 *μ*m filter (Millipore) and used to determine free PAs by benzoylation, and derivatives analysed by HPLC (UnicamCrystal-200, UK). A 10 *μ*L of filtered supernatant was used for this purpose. The elution system consisted of MeOH/H_2_O (64 : 36) solvent, running isocrati0cally with a flow rate of 0.8 mL min^−1^ through a reversed-phase column (LiChroCart 250-4.5 *μ*m) and detection was based on UV absorbance at 254 nm. PAs were identified and quantified by comparing retention times and peak areas with authentic standards (Sigma–Aldrich, USA). Results were expressed as nmol g^−1^ fresh weight.

### 2.5. Statistical Analysis

All data were subjected to two-way analysis of variance (ANOVA) performed with the SAS 9.1.3 service pack 4 software (SAS Institute, Cary, NC, USA), and the means were separated by the least significant difference (LSD) test at P ≤0.05.

## 3. Results

### 3.1. MDA Content and CI Index

Mean comparisons showed that the MDA content increased significantly during storage. Generally, the warming resulted in a significantly lower MDA content compared to untreated fruit. Moreover, the mean value at the end of storage was significantly higher than the time of interruption (Table 1).

The amount of MDA in the peel of control fruit was nearly similar to the harvesting time until the 25th day of cold storage, and there was no significant difference between the cold-stored fruit sampled on the 15th and 25th days of storage. Subsequently, there was a significant increasing trend along with storage time. Fruit warming at 15th and 45th days instantly reduced the MDA content of the peel compared to the controls (Table 2). On the other hand, at the end of the storage period, the fruit treated at interruption dates had significantly lower MDA (Table 2) and CI index (Table 3) compared to the controls with the lowest levels recorded for the fruit treated on the 15th and 25th days.

### 3.2. FAs Modifications

#### 3.2.1. SFAs, Mono and Poly UFAs

**Figure 1:**
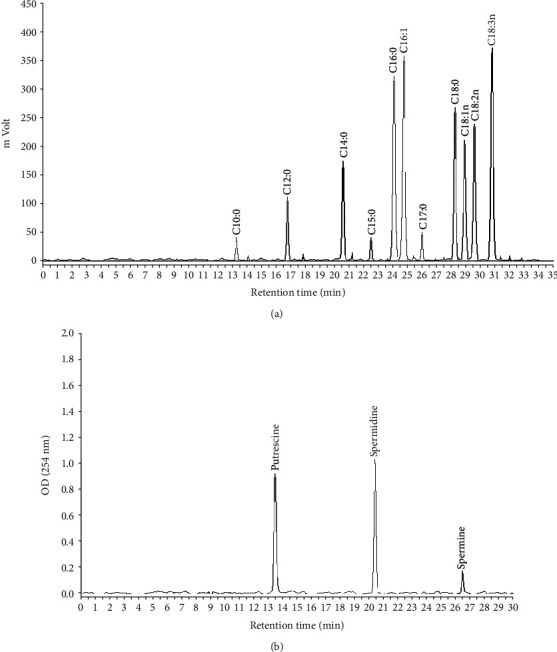
GC chromatogram of Fatty Acids (a), and HPLC chromatogram of Polyamines (b) in pomegranate peel. C10: Capric acid; C12: Lauric acid; C14: Myristic acid; C15: Pentadecylic acid; C16: Palmitic acid; C17: Margaric acid; C18: Stearic acid; C16:1: Palmitoleic acid; C18:1: Oleic acid; C18:2: Linoleic acid and C18:3 Linolenic acid.

The following FAs have been identified and quantified in the peel samples (Figure 1(a) and Table 2): capric acid (C10), lauric acid (C12), myristic acid (C14), pentadecylic acid (C15), palmitic acid (C16), margaric acid (C17) and stearic acid (C18) as saturated (SFAs); palmitoleic acid (C16:1) and oleic acid (C18:1) as monounsaturated (MUFAs); and linoleic acid (C18:2) and linolenic acid (C18:3) as polyunsaturated (PUFAs) ones.

#### 3.2.2. Total SFAs (Membrane Saturation Index)

The fruit had significantly higher total SFAs at the last interruption time (45th day) of storage than other times. In addition, statistically lower mean values were recorded for the warmed fruit and those sampled at the interruption dates compared to the untreated fruit and those sampled at the end of the experiment, respectively (Table 1).

**Figure 2:**
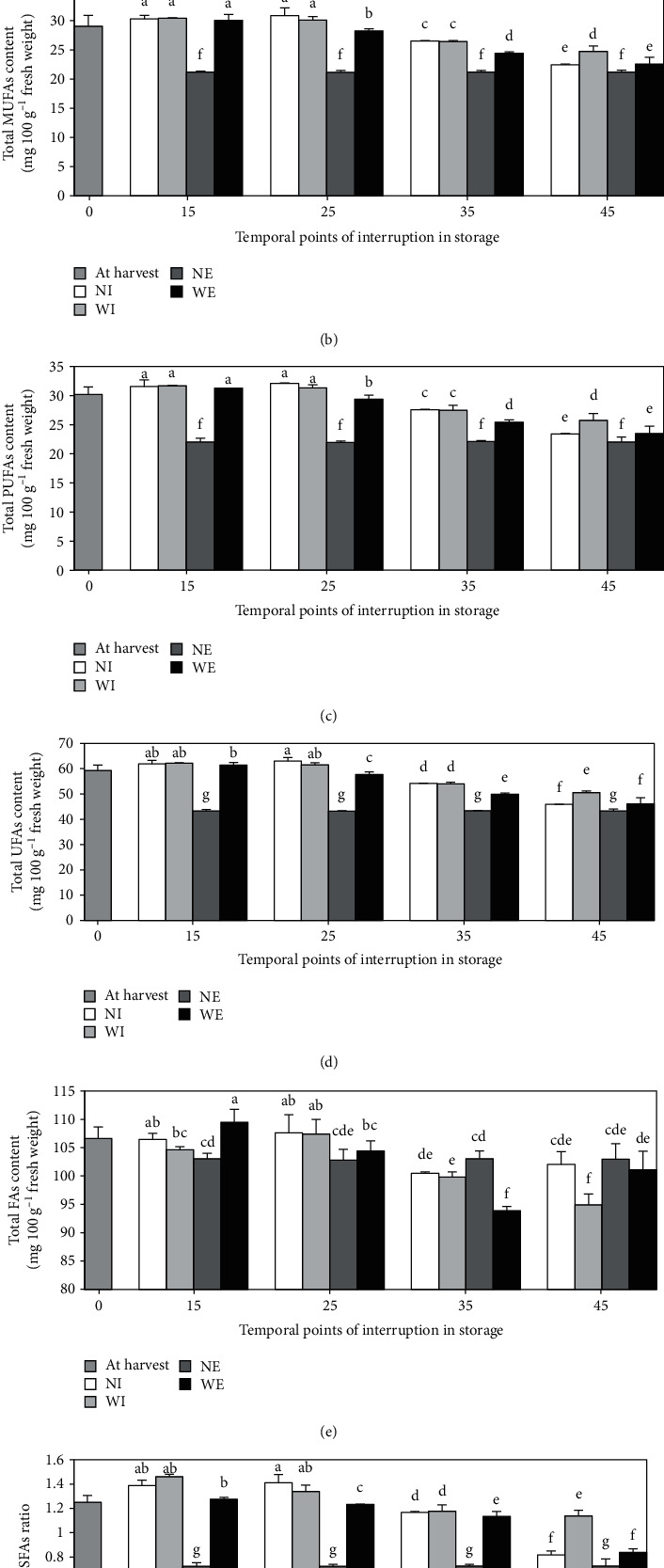
Modifications in the total content of saturated (a), monounsaturated (b), polyunsaturated (c), and unsaturated (d) fatty acids, total fatty acids (e), and unsaturated/saturated fatty acids ratio (f) in pomegranate fruit peel during 70 days storage (at 2 ± 0.5°C and 90 ± 5% RH). Intermittent warming was conducted in the form of a single warming period (1 day at 20°C with 70% RH) before returning the treated fruit to storage. Temporal points of interruption were 15th, 25th, 35th, or 45th days of storage. WI stands for warmed fruit which were immediately sampled after warming on the date of interruption, NI stands for not warmed fruit which were sampled on the date of interruption, WE stands for warmed fruit, which were returned to storage and sampled at the end of the storage period, and NE stands for not warmed fruit sampled at the end of the storage period. Data are means of 3 replicates ± SD. All statistical differences (by LSD test, P ≤0.05) throughout the storage period are shown in different letters.

Up to 35 days of cold storage, the total SFAs did not change. Subsequently, a significant increasing trend was detected, with the highest amount recorded at the end of storage. The instant effect of warming on the membrane saturation index was detected only on the fruit treated on the 45th day, leading to a statistical decrease in the index compared to the control. However, treating the fruit at any time of interruption in storage resulted in significantly lower total SFAs recorded at the end of the experiment compared to the controls (Figure 2(a)).

#### 3.2.3. Total MUFAs, PUFAs, and UFAs (Membrane Unsaturation Index)

The results showed a significant decrease in all unsaturation indices during cold storage. On the other hand, the mean values of the warmed fruit and fruit immediately sampled at interruption dates were significantly higher than those of the untreated fruit and fruit sampled at the end of the cold storage period, respectively (Table 1).

Total MUFAs, PUFAs, and therefore membrane unsaturation index increased to the 25th day of storage, even more than the harvesting time. Subsequently, a significant decreasing trend was identified until the end of storage. By warming the fruit on the 45th day of cold storage, all unsaturation indices were increased significantly, even though at the end of the experiment, the fruit treated at any time during the storage period had significantly higher levels of MUFAs, PUFAs and UFAs than the controls (Figures 2(b), 2(c) and 2(d)).

#### 3.2.4. Total FAs

Mean comparisons showed that the total FAs content at the first and second interruption dates was significantly higher than at the later dates, with no significant difference between the fruit sampled at the 15th and 25th or 35th and 45th days. Generally, the treated fruit had significantly lower total FAs than the untreated fruit, and there was no significant difference between the fruit sampled at interruption dates or at the end of the experiment (Table 1).

On the one hand, the total FAs increased to the 25th day of storage, even more than the harvesting time. On the other hand, the index subsequently decreased significantly, without a statistical difference between the fruit sampled on the 35th day and later. Fruit warming at interruption dates had no immediate impact on total FAs content except on the 45th day, which resulted in a significant decrease in the index compared to the control. Moreover, only fruit treated during the first month had higher total FAs compared to controls at the end of the storage period. From this point of view, there was a significant difference between the fruit treated on the 15th day and the control fruit at the end of the experiment (Figure 2(e)).

#### 3.2.5. UFAs/SFAs Ratio (Membrane Integrity Index)

It was observed a significant decreasing trend in the UFAs/SFAs ratio with progress in the storage period. In addition, a significantly higher mean value was found for treated fruit and fruit immediately sampled at interruption dates compared to untreated fruit and those sampled at the end of the storage period (Table 1).

The membrane integrity index improved to the 25th day of storage, even better than the harvesting time. Subsequently, a significant decreasing trend was detected up to the end of storage. On the other hand, at the end of the storage period, all fruit treated at interruption dates had a statistically higher UFAs/SFAs ratio compared to controls (Figure 2(f)).

### 3.3. PAs Modifications

The three main polyamines, putrescine (Put), spermidine (Spd), and spermine (Spm) in their free forms, were identified and quantified in pomegranate peel (Figure 1(b)).

#### 3.3.1. Put Content

Mean comparisons showed that the advancement in storage from the 25th day to the end was followed by a significant decrease in the concentration of Put. Treated fruit had significantly higher content of Put than untreated fruit. In addition, the amount of Put was statistically lower at the end of storage than during storage (Table 4).

**Figure 3:**
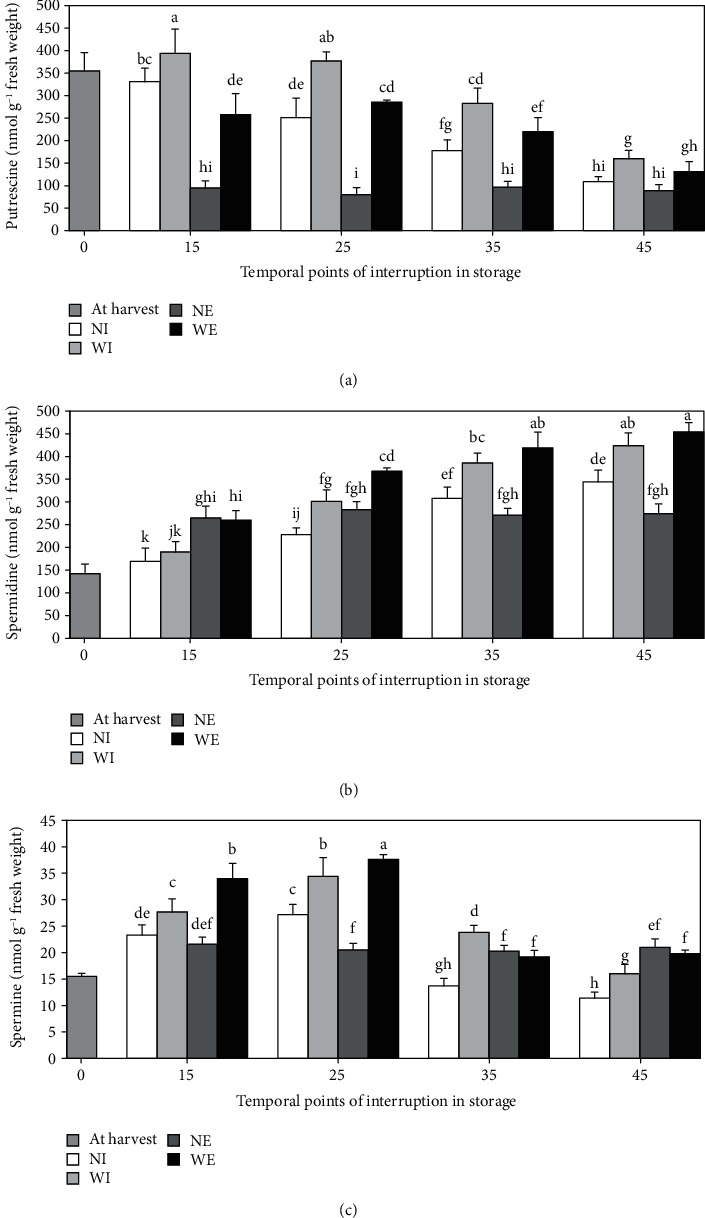
Modifications in the content of putrescine (a), spermidine (b), and spermine (c) in pomegranate fruit peel during 70 days storage (at 2 ± 0.5°C and 90 ± 5% RH). Intermittent warming was conducted in the form of a single warming period (1 day at 20°C with 70% RH) before returning the treated fruit to storage. Temporal points of interruption were 15th, 25th, 35th, or 45th days of storage. WI stands for warmed fruit which were immediately sampled after warming on the date of interruption, NI stands for not warmed fruit which were sampled on the date of interruption, WE stands for warmed fruit, which were returned to storage and sampled at the end of the storage period, and NE stands for not warmed fruit sampled at the end of the storage period. Data are means of 3 replicates ± SD. All statistical differences (by LSD test, P ≤0.05) throughout the storage period are shown in different letters.

During cold storage, the content of Put decreased significantly and continuously until the 45th day, with no statistical difference between the 45th and 70th days. Warming at interruption dates resulted in an immediate increase and higher final contents compared to controls, except for the fruit treated on the 45th day which had the final amount of Put the same as the control (Figure 3(a)).

#### 3.3.2. Spd Content

According to the mean comparison, the concentration of Spd increased significantly as the storage time progressed. In addition, the treated fruit had statistically higher levels of Spd than the untreated fruit and the amount of Spd at the end of storage was significantly higher than what was recorded during that period (Table 4).

Statistically, the internal concentration of Spd was continuously increased until the 35th day of cold storage, with no differences between the fruit sampled on the 35th and 45th days. Subsequently, a significant decrease in Spd content was recorded for the last 25 days of cold storage. Warming at all interruption dates, except for the 15th day, resulted in a significant instant increase and a statistically higher final value of Spd at the end of storage compared to the control fruit (Figure 3(b)).

#### 3.3.3. Spm Content

Mean comparisons showed that more Spm level was detected in the fruit sampled on the 25th day of storage and those sampled on the 15th, 35th, and 45th days were at the next position, respectively, and all differences were significant. Generally, the warming resulted in statistically higher Spm content and the value detected at the end of storage was significantly higher than that measured at the time of interruption (Table 4).

Modifications in the concentration of Spm during storage could be categorized into three different parts: an increase up to the 25th day, subsequent decrease up to the 45th day, and then increase up to the end of storage. Most of the differences were almost significant. Warming during storage immediately led to a significant increase in the content of Spm. Although only fruits treated on the 15th and 25th days had statistically more Spm than controls at the end of the storage period, the other time-treated fruit were the same as the controls. The fruit sampled on the 25th day had a statistically higher endogenous content of Spm compared to other different time-sampled fruit and had a significantly higher instant increase and final content of Spm in response to treatment than the fruit treated at the other interruption dates (Figure 3(c)).

## 4. Discussion

### 4.1. MDA Content and CI Index

The investigation of changes in the MDA content (Table 2) revealed that lipid peroxidation increased after one month of chilling stress. Examining the physiological changes and the incidence of chilling injury in the same storage condition as we did earlier, Taghipour et al. [8] showed that pomegranate fruit cv. Rabab-e-Neyriz could be stored in cold temperature without significant CI for up to 30 days. From this point of view, our finding was consistent with them. At the end of the storage period, all different time-treated fruit had a significantly lower MDA and CI index compared to the controls, with the lowest levels recorded for the fruit treated on the 15th and 25th days (Tables 2 and 3). In accordance with our finding, Taghipour et al. [8] suggested that the warming should be carried out during the first month of cold storage, predicting it with the possible desired effect of extending the shelf life of this commercial cultivar. These data suggested IW's prominent efficacy in alleviating the incidence of pomegranate CI at low temperatures, which part of the related mechanisms are discussed based on our findings in the following parts of discussion.

### 4.2. FAs Modifications

It has been accepted that chilling-resistant subtropical and tropical horticultural crops have greater levels of UFAs and UFAs/SFAs ratio in cell membrane lipids [22, 23]. Higher levels of UFAs lead to higher membrane fluidity, identified as part of low-temperature tolerance mechanisms [24–26]. It means that the intensity of the lipid phase shift from flexible liquid crystalline to solid gel is lower in the membrane with greater fluidity, thus maintaining the membrane permeability and greater tolerance to low temperatures [5, 25]. For example, it has been indicated that chilling-tolerant cultivars of loquat fruit have a greater linoleic (C18:2) and linolenic (C18:3) acid content and lower palmitic (C16) and stearic acid (C18) concentrations, resulting in a greater UFAs/SFAs ratio [27]. On the one hand, higher activity of phospholipase D (PLD) and lipoxygenase (LOX) enzymes could be liable for UFAs degradation at chilling temperatures leading to decreased cell membrane integrity and increased adverse effects of CI [28]. On the other hand, non-enzymatic oxidation of UFAs by reactive oxygen species (ROS) associated with MDA production could result in reduced membrane integrity and increased adverse effects of CI [29]. The integrity of the membrane plays a crucial role in fruit pericarp browning as one of the CI symptoms [29].

Results indicated that the values of all SFAs (Table 2) and the membrane saturation index (Figure 2(a)) of the fruit sampled at any time of interruption up to the 35th day of cold storage were statistically the same and, in most cases, even lower than the harvest time value. Afterwards, as shown in Table 2, there was a significant increasing trend for all SFAs up to the 45th day of storage, which was continued only for palmitic acid during the last 25 days of the cold storage period. On the other hand, all MUFAs and PUFAs have risen up to the 25th day of storage, even more than the harvest time. However, a declining trend with significant differences between the fruit sampled on the 25th, 35th, and 45th days was subsequently founded, the latter having statistically the same contents of all MUFAs and PUFAs as detected at the end of the storage period. These data indicated that the fruit had significant changes in peel fatty acids following the expected adverse effects of CI on cell membrane integrity after approximately 1 month of cold storage. The stability of the membrane saturation index up to the 35th day (Figure 2(a)) and the stability of the unsaturation index up to the 25th day (Figure 2(d)), without an increase in the MDA level during the first month of storage (Table 2), could be associated with low-temperature fruit acclimatization to prevent chilling injury. This is similar to what was reported by Antunes and Sfakiotakis [30], as a major increase in the UFAs/SFAs ratio during the first days of kiwifruit storage as a response to tissue adaptation to new stress storage conditions.

Transferring cold-stored fruit to an elevated temperature and returning it to the previous temperature has been reported to regulate and readjust the metabolic processes leading to improved UFAs synthesis. In other words, the warming appears to result in an increase in the synthesis and elongation of the SFAs chains, which can act as a substrate for UFAs synthesis and increase the degree of unsaturation of the cell membrane after returning to cold temperature [6, 7, 10]. It has been noted, for instance, that peach and cucumber warming caused an increase in C18, while subsequent exposure to cold temperature resulted in an increase in C18:1, C18:2 and C18:3. It was suggested that IW could result in the enhanced amount of C18 as a prerequisite for UFAs synthesis, thus improving the degree of unsaturation in membrane lipids [6]. Rapid changes in membrane lipids with altered temperatures have also been reported in soybean roots. As the temperature was raised, C16 and C18 increased, but C18:1, C18:2, and C18:3 decreased in plasma membranes and mitochondrial membrane; the reverse trend happened as the temperature was lowered [31].

Moreover, the data collected at the end of storage showed that fruit treated for up to 35 days of cold storage had significantly higher amounts of all MUFAs and PUFAs (Table 2) and, as a result, higher levels of total MUFAs, PUFAs and UFAs (Figures 2(b), 2(c) and 2(d)) compared to control. Notably, in the fruit treated on the 15th day, these indices remained unchanged until the end of the experiment. Indeed, the fruit treated on the 45th day had a significant immediate increase in all MUFAs, PUFAs (Table 2) and total MUFAs, PUFAs and UFAs (Figures 2(b), 2(c) and 2(d)), leading to constant higher values compared to the control at the end of the storage period. On the other hand, the results showed that treatment at any time of interruption during storage did not result in immediate changes in the concentration of SFAs (Table 2) and the membrane saturation index (Figure 2(a)), except for a significant reduction in the indices on the 45th day. It was concluded that the statistically higher levels of UFAs and the unsaturation rates recorded at the end of the cold-warm-cold period could be mainly independent of an instant increase in SFAs in response to warming as a mandatory prerequisite. It could well be associated to the warm-induced mechanisms responsible for protecting UFAs at chilling temperatures. The warming on the 45th day led to an immediate and statistical increase in all UFAs, and a decrease in all SFAs and MDA content simultaneously (Table 2), indicating the possibility of a direct impact of treatment as an increase in UFAs, UFAs/SFAs ratio and a reduction in the peroxidation rate of UFAs. It means that the desaturation of SFAs could be a direct result of warming. This finding contrasts with the previous suggestions that the desaturation of SFAs is triggered by the return of the treated fruit to low temperatures [6, 7, 10]. The activity of LOX and PLD enzymes as a direct effect of warming may also be likely to decrease. To be proved, this claim needs to be studied.

### 4.3. PAs Modifications

Based on some of the scientific evidence, the protective role of PAs in alleviating different types of stress in plants has been proposed. Reducing endogenous polyamines by applying their biosynthesis inhibitors [32–34] or by using polyamine biosynthesis mutants [35, 36] has been shown to result in increased sensitivity to stress and increased injury to affected tissues. Moreover, exogenous application of polyamines before or during stress [33, 37–40] could lead to an increase in their endogenous content, leading to a reduction in stress-related injuries to varying degrees, depending on a number of factors, such as individual polyamines, plant species or cultivars, type of stress and its duration and intensity [41].

The relationship between CI and damage to the membranes has been proven [5]. On the one hand, it has been suggested that PAs, due to their antioxidant activity, are potential for membrane binding via interactions with phospholipids [12, 42] and triggering enzymatic antioxidant activities [41, 43–45] could be responsible for lower changes in membrane fluidity and solute leakage [13], and play an important protective role in the safety of membranes under conditions of stress [14]. On the other hand, PAs rise concomitantly with CI incidence has been reported in chilling-sensitive horticultural crops [46], but it remains unclear whether PAs rise is due to chilling stress or a defensive mechanism against CI [47]. For example, increases in the concentration of Put in conjunction with CI incidence for lemon, orange, lime, grapefruit, pepper, zucchini, and pepino fruit have been detected [48–52]. Moreover, it has been reported that IW promotes the production of PAs in treated tissues during the warming phase [11]. It was therefore of great interest for the authors to explore possible associations between the incidence of CI, natural changes in endogenous PAs and their modifications in response to IW treatment of pomegranate fruit as a sensitive commodity.

Expression of PA biosynthetic genes under different stresses could be regulated in a disparate manner, including immediate induction with continuous increase or minor changes during stress periods, or induction only in response to certain stress periods [53–56]. In addition, different cultivars of the same species might also have different patterns of PAs under stress [41]. The endogenous Spm in the control fruit was significantly increased during the first month of cold storage (Figure 3(c)). This could be related to the activation of the PA biosynthesis pathway as part of the hypothesized ability of the ‘Rabab-e-Neyriz' fruit to acclimate to stress. By increasing the internal content of Spd alongside the advancement in storage time (Figure 3(b)), it was concluded that Spd could play a more prominent role in alleviating the incidence of CI by extending the exposure time to chilling temperature.

Existing literature shows that postharvest techniques associated with high levels of PAs reduce CI in many sensitive horticultural commodities. For example, exogenous prestorage applications of PAs could decrease CI in commodities such as apple [57], zucchini [58], and mango [59, 60]. Likewise, pomegranate pre-storage treatment by Put or Spd significantly reduced the incidence of CI at chilling temperature; this effect was associated with enhanced endogenous PAs [47]. In addition to IW, the pre-storage hot water dip is known as the main method for postharvest heat treatment of horticultural commodities, which could reduce the incidence of CI associated with rises in endogenous PAs in sensitive crops [48, 61–66]. Accordingly, by prestorage hot water dip (at 45°C for 4 min), Mirdehghan et al. [21] reported a reduction in CI and an increase in PAs in the peel of the pomegranate with such a value were always higher than in control fruit, even more than that measured at harvest. One notable finding was an instantly and statistically enhanced biosynthesis of Put and Spm in fruit treated during the first month of storage compared to control or later treated fruit (Figures 3(a) and 3(c)). In contrast to the control fruit, in the case of fruit treated on the 15th or 25th day, Spm increased steadily and significantly until the end of the storage period (Figure 3(c)). It was concluded that the rate of immediate increase and the final content of Spm in response to treatment was time-dependent and that the warming treatment was assumed to have a synergistic effect with the inherent potential of Spm biosynthesis in the fruit peel. On the one hand, only fruit treated during the first month of storage had significantly higher final Spm content than the control fruit (Figure 3(c)). On the other hand, postponing the warming treatment to more than one month of cold storage significantly lowered the rate of treatment effect on Put biosynthesis and its final value (Figure 3(a)). These findings could emphasize the importance of performing IW treatment at the right time during storage to achieve optimum performance in extending the shelf life of the treated fruit. By warming on or after the 25th day, Spd was also immediately and significantly increased, resulting in statistically higher levels both at interruption dates and at the end of the storage period in the treated fruit compared to the control fruit (Figure 3(b)). As the storage period increased, the content of Put decreased significantly in the control fruit. Furthermore, the different time-treated fruit had a final Put content lower than that recorded after harvest (Figure 3(a)). Accumulation of Put is a general reaction to stress [43], although it could be converted into Spd via spermidine synthase (SPDS, EC 2.5.1.16) [41]. It was likely that part of the continuous decrease in the content of Put in the peel of cold-stored fruit was due to the conversion to Spd. In the same way, it could be assumed that part of the increased content of Put in the treated fruit has also been transformed into Spd, with a higher rate of transformation coinciding with the progress in storage time. This hypothesized mechanism could contribute to the significantly higher levels of Spd detected in fruit treated on or after the 25th day compared to control fruit both at interruption dates and at the end of storage time.

As stated earlier, it was established from the other part of our research [17] that IW, as a single cold-warm-cold cycle, could have beneficial effects on the cold storage of ‘Rabab-e-Neyriz' pomegranate by inducing enzymatic and non-enzymatic antioxidant reactions in the fruit peel. It was concluded that maintaining higher levels of antioxidant activity is likely to be responsible for reducing lipid peroxidation following warming. It was founded that fruit treated for up to 35 days had significantly higher final activity of superoxide dismutase (SOD), peroxidase (POD), and ascorbate peroxidase (APX) than controls at the end of storage, with earlier warming resulting in higher activity. Furthermore, peel browning was not related to POD activity and this enzyme had only a beneficial antioxidant function. By investigating the mode of change in the content of 13 phenolic acids (as non-enzymatic antioxidants), it was also founded that the total phenolic content of the fruit treated at any time of interruption was statistically higher compared to the control, with a value higher than the time of harvest for the fruit treated during the first month of cold storage. In addition to being an important protective mechanism against CI incidence in fruit, higher total phenolic content can be regarded as an indicator of natural antioxidant sources available in the food industry.

On the basis of the overall findings of our research, it can be asserted that a single warming period at the right time during cold storage of pomegranate (before irreversible chilling damage occurs) triggers multiple mechanisms and physiological responses in fruit peel which are significantly responsible for alleviating the severity of chilling damage to this valuable horticultural commodity.

## 5. Conclusions

Using one cycle of IW by warming the cold-stored pomegranate fruit on the 15th day of storage led to an immediate and significant increase in the endogenous content of Spm and Put, followed by a continuous increase in the level of Spm and a higher level of Put up to the end of storage for treated fruit compared to control fruit. Furthermore, the cell membrane integrity index remained unchanged for the treated fruit until the end of the storage period. It was concluded that warming was likely to induce the protective mechanisms responsible for preserving UFAs from peroxidation, including modifications to endogenous PAs as membrane safety agents. The beneficial effect of the treatment was adversely affected by postponing during storage. Our findings could be crucial for industrial IW applications. It is highly recommended that the efficacy of a single warming period during cold storage in maintaining the postharvest quality of other perishable horticultural crops with beneficial effects on human health be evaluated. It is predictable that the use of this safe and nonchemical postharvest treatment will result in a longer period of time for consumers to have access to high-quality, health-promoting horticultural crops for use.

## Figures and Tables

**Table 1:** Effects of experimental factors on the malondialdehyde content; total saturated, monounsaturated, polyunsaturated, and unsaturated fatty acids; total fatty acids and the ratio of unsaturated/saturated fatty acids in the peel of cold-stored pomegranate.

	MDA (*μ*mol g^−1^ fresh weight)	Total SFAs (mg 100 g^−1^ fresh weight)	Total MUFAs (mg 100 g^−1^ fresh weight)	Total PUFAs (mg 100 g^−1^ fresh weight)	Total UFAs (mg 100 g^−1^ fresh weight)	Total FAs (mg 100 g^−1^ fresh weight)	UFAs/SFAs
Temporal point of interruption in storage (day)							
15	19.70 ± 0.072^d^	48.76 ± 0.048^b^	28.00 ± 0.028^a^	29.15 ± 0.030^a^	57.15 ± 0.058^a^	105.90 ± 0.019^a^	1.21 ± 0.002^a^
25	21.80 ± 0.058^c^	49.27 ± 0.045^b^	27.60 ± 0.028^a^	28.70 ± 0.029^a^	56.30 ± 0.057^b^	105.57 ± 0.021^a^	1.18 ± 0.002^b^
35	28.26 ± 0.035^b^	49.00 ± 0.046^b^	24.65 ± 0.016^b^	25.66 ± 0.016^b^	50.31 ± 0.031^c^	100.26 ± 0.025^b^	1.05 ± 0.001^c^
45	32.37 ± 0.024^a^	53.84 ± 0.044^a^	22.74 ± 0.010^c^	23.68 ± 0.011^c^	46.42 ± 0.020^d^	99.32 ± 0.028^b^	0.88 ± 0.001^d^
Warming regime							
Warming	22.50 ± 0.012^b^	46.59 ± 0.006^b^	27.12 ± 0.005^a^	28.24 ± 0.005^a^	55.36 ± 0.010^a^	101.95 ± 0.010^b^	1.20 ± 0.001^a^
Not warming	28.57 ± 0.016^a^	53.84 ± 0.012^a^	24.37 ± 0.007^b^	25.36 ± 0.007^b^	49.73 ± 0.014^b^	103.57 ± 0.005^a^	0.96 ± 0.001^b^
Sampling time							
Immediately	20.54 ± 0.013^b^	46.33 ± 0.007^b^	27.73 ± 0.005^a^	28.87 ± 0.006^a^	56.59 ± 0.011^a^	102.91 ± 0.008^a^	1.24 ± 0.001^a^
At the end of storage	30.53 ± 0.012^a^	54.10 ± 0.011^a^	23.76 ± 0.006^b^	24.73 ± 0.006^b^	48.50 ± 0.012^b^	102.60 ± 0.008^a^	0.92 ± 0.001^b^

Data are means of replicates (*n* =12 for each interruption date in storage and 24 for each warming regime and sampling date) ± standard error. Fruit were stored for 70 days at 2 ± 0.5°C and 90 ± 5% relative humidity. Warming was performed as 1 day at 20°C with 70% relative humidity. MDA: malondialdehyde; SFAs: saturated fatty acids; MUFAs: monounsaturated fatty acids; PUFAs: polyunsaturated fatty acids; UFAs: unsaturated fatty acids; FAs: fatty acids and UFAs/SFAs: unsaturated/saturated fatty acids ratio. For each experimental factor and evaluated index, means followed by the same letter are not significantly different by least significant difference (LSD) test, P <0.05.

**Table 2:** The content of malondialdehyde and fatty acids in pomegranate fruit peel under one intermittent warming cycle during 70 days of cold storage.

	MDA (*μ*mol g^−1^ fresh weight)	SFAs (mg 100 g^−1^ fresh weight)							MUFAs (mg 100 g^−1^ fresh weight)		PUFAs (mg 100 g^−1^ fresh weight)	
		C10	C12	C14	C15	C16	C17	C18	C16:1	C18:1	C18:2	C18:3
At harvest	18.60 ± 0.74	1.46 ± 0.11	5.36 ± 0.20	8.67 ± 0.45	1.49 ± 0.13	16.39 ± 0.66	1.92 ± 0.27	12.10 ± 1.53	17.78 ± 1.07	11.26 ± 1.63	12.44 ± 0.67	17.75 ± 1.47
Treatments												
15NI	13.44 ± 0.17^g^	1.35 ± 0.09^bc^	5.02 ± 0.06^cd^	8.18 ± 0.14^cd^	1.36 ± 0.14^bc^	15.47 ± 0.37^de^	1.83 ± 0.9^bc^	11.37 ± 0.49^cde^	18.56 ± 0.49^a^	11.74 ± 0.60^ab^	12.99 ± 0.77^a^	18.56 ± 0.59^a^
15WI	10.22 ± 0.16^h^	1.30 ± 0.19^c^	4.77 ± 0.21^d^	7.81 ± 0.56^d^	1.30 ± 0.13^c^	14.76 ± 0.85^e^	1.73 ± 0.16^c^	10.85 ± 0.75^e^	18.63 ± 0.77^a^	11.79 ± 0.69^ab^	13.04 ± 0.71^a^	18.65 ± 0.63^a^
15NE	35.93 ± 1.69^a^	1.82 ± 0.07^a^	6.72 ± 0.19^a^	10.98 ± 0.41^a^	1.80 ± 0.05^a^	20.78 ± 0.59^a^	2.41 ± 0.16^a^	15.29 ± 0.39^a^	12.97 ± 0.33^e^	8.25 ± 0.37^g^	9.13 ± 0.22^d^	12.92 ± 0.46^d^
15WE	19.22 ± 0.58^e^	1.48 ± 0.21^b^	5.40 ± 0.38^cd^	8.83 ± 0.58^c^	1.47 ± 0.13^bc^	16.70 ± 0.52^c^	1.96 ± 0.17^b^	12.27 ± 0.52^c^	18.40 ± 0.56^a^	11.66 ± 0.46^ab^	12.89 ± 0.82^a^	18.41 ± 0.80^a^
25NI	15.21 ± 0.57^fg^	1.37 ± 0.13^bc^	5.01 ± 0.44^cd^	8.21 ± 0.79^cd^	1.37 ± 0.16^bc^	15.50 ± 1.06^de^	1.82 ± 0.12^bc^	11.40 ± 0.91^cde^	18.90 ± 1.01^a^	11.95 ± 0.69^a^	13.21 ± 0.68^a^	18.88 ± 0.71^a^
25WI	16.74 ± 0.62^f^	1.40 ± 0.13^bc^	5.16 ± 0.41^cd^	8.45 ± 0.44^cd^	1.40 ± 0.17^bc^	15.94 ± 0.69^cd^	1.87 ± 0.11^bc^	11.72 ± 0.88^cde^	18.45 ± 0.28^a^	11.67 ± 0.60^ab^	12.91 ± 0.90^a^	18.44 ± 0.50^a^
25NE	35.34 ± 1.19^a^	1.81 ± 0.08^a^	6.69 ± 0.22^a^	10.99 ± 0.33^a^	1.83 ± 0.09^a^	20.74 ± 0.66^a^	2.42 ± 0.11^a^	15.18 ± 0.40^a^	12.98 ± 0.47^e^	8.20 ± 0.33^g^	9.03 ± 0.30^d^	12.95 ± 0.25^d^
25WE	19.95 ± 0.25^e^	1.36 ± 0.04^bc^	5.01 ± 0.29^cd^	8.74 ± 0.08^c^	1.49 ± 0.02^b^	16.31 ± 0.09^cd^	1.92 ± 0.04^bc^	11.97 ± 0.35^cd^	17.22 ± 0.23^b^	11.03 ± 0.13^bc^	12.31 ± 0.09^a^	17.09 ± 0.58^b^
35NI	23.84 ± 1.01^d^	1.39 ± 0.09^bc^	5.54 ± 0.35^bc^	8.46 ± 0.38^cd^	1.40 ± 0.09^bc^	15.97 ± 0.45^cd^	1.87 ± 0.09^bc^	11.75 ± 0.52^cde^	16.25 ± 0.38^c^	10.28 ± 0.36^cd^	11.36 ± 0.55^b^	16.23 ± 0.51^b^
35WI	24.11 ± 0.81^d^	1.38 ± 0.07^bc^	5.15 ± 0.41^cd^	8.45 ± 0.37^cd^	1.39 ± 0.10^bc^	15.96 ± 0.76^cd^	1.86 ± 0.06^bc^	11.69 ± 0.62^cde^	16.18 ± 0.45^c^	10.24 ± 0.61^cde^	11.32 ± 0.42^b^	16.17 ± 0.42^b^
35NE	35.72 ± 1.22^a^	1.81 ± 0.07^a^	6.71 ± 0.34^a^	10.97 ± 0.40^a^	1.84 ± 0.10^a^	20.70 ± 0.48^a^	2.43 ± 0.10^a^	15.27 ± 0.49^a^	12.96 ± 0.53^e^	8.26 ± 0.31^g^	9.06 ± 0.24^d^	13.07 ± 0.31^d^
35WE	29.36 ± 0.86^c^	1.37 ± 0.09^bc^	4.93 ± 0.48^cd^	8.09 ± 0.46^cd^	1.37 ± 0.09^bc^	15.26 ± 0.53^de^	1.78 ± 0.08^bc^	11.22 ± 0.42^de^	14.95 ± 0.40^d^	9.48 ± 0.36^ef^	10.47 ± 0.31^bc^	14.97 ± 0.65^c^
45NI	33.21 ± 1.99^b^	1.71 ± 0.07^a^	6.31 ± 0.51^a^	10.33 ± 0.41^ab^	1.72 ± 0.07^a^	19.52 ± 0.56^b^	2.30 ± 0.21^a^	14.30 ± 0.45^ab^	13.74 ± 0.72^e^	8.72 ± 0.61^fg^	9.64 ± 0.62^cd^	13.77 ± 0.73^d^
45WI	27.55 ± 1.31^c^	1.35 ± 0.05^bc^	4.98 ± 0.39^cd^	8.17 ± 0.49^cd^	1.36 ± 0.05^bc^	15.43 ± 0.52^de^	1.81 ± 0.07^bc^	11.34 ± 0.55^cde^	15.14 ± 0.54^d^	9.59 ± 0.41^de^	10.60 ± 0.64^b^	15.15 ± 0.55^c^
45NE	35.87 ± 2.46^a^	1.82 ± 0.08^a^	6.70 ± 0.60^a^	10.97 ± 0.69^a^	1.82 ± 0.10^a^	20.73 ± 1.32^a^	2.43 ± 0.26^a^	15.24 ± 0.73^a^	12.97 ± .67^e^	8.22 ± 0.64^g^	9.08 ± 0.32^d^	12.98 ± 0.50^d^
45WE	32.85 ± 2.07^b^	1.66 ± 0.07^a^	6.18 ± 0.42^ab^	10.11 ± 0.50^b^	1.68 ± 0.08^a^	19.10 ± 0.59^b^	2.24 ± 0.09^a^	14.05 ± 0.40^b^	13.82 ± 0.81^e^	8.76 ± 0.38^fg^	9.68 ± 0.62^cd^	13.83 ± 0.65^d^
LSD	2.109	0.161	0.649	0.783	0.178	1.112	0.200	0.994	0.858	0.793	0.916	0.928

Fruit were stored at 2 ± 0.5°C and 90 ± 5% relative humidity. Intermittent warming cycle was 1 day at 20°C with 70% relative humidity before returning the treated fruit to storage. Temporal points of interruption were 15th, 25th, 35th, or 45th days of storage. WI stands for warmed fruit which were immediately sampled after warming on the date of interruption, NI stands for not warmed fruit which were sampled on the date of interruption, WE stands for warmed fruit, which were returned to storage and sampled at the end of the storage period, and NE stands for not warmed fruit sampled at the end of the storage period. MDA: malondialdehyde; SFAs: saturated fatty acids; MUFAs: monounsaturated fatty acids; PUFAs: polyunsaturated fatty acids; C10: Capric acid; C12: Lauric acid; C14: Myristic acid; C15: Pentadecylic acid; C16: Palmitic acid; C17: Margaric acid; C18: Stearic acid; C16:1: Palmitoleic acid; C18:1: Oleic acid; C18:2: Linoleic acid and C18:3: Linolenic acid. Data are means of 3 replicates ± standard deviation. Means within a column followed by the same letter are not significantly different by least significant difference (LSD) test, P <0.05.

**Table 3:** Differences in chilling injury index between control and intermittently warmed fruit after 70 days of cold storage and additional 3 days shelf life at 20°C.

	Warming day during storage				
	C	15	25	35	45
CI index	0.48 ± 0.04^a^	0.18 ± 0.04^d^	0.21 ± 0.03^d^	0.27 ± 0.03^c^	0.33 ± 0.02^b^

Fruit were stored at 2 ± 0.5°C and 90 ± 5% relative humidity. Intermittent warming was conducted in the form of a single warming period (1 day at 20°C with 70% relative humidity) before returning the treated fruit to storage. Temporal points of interruption were 15th, 25th, 35th, or 45th days of storage. CI: Chilling injury; and C: control fruit. Data are means of 3 replicates ± standard deviation. Means with the same letter are not significantly different by least significant difference (LSD) test, P <0.05.

**Table 4:** Effects of experimental factors on the content of polyamines (PAs) in the peel of cold-stored pomegranate.

	Put (nmol g^−1^ fresh weight)	Spd (nmol g^−1^ fresh weight)	Spm (nmol g^−1^ fresh weight)
Temporal point of interruption in storage (day)			
15	269.50 ± 0.84^a^	221.00 ± 0.34^d^	26.65 ± 0.04^b^
25	248.38 ± 0.79^a^	295.00 ± 0.38^c^	29.94 ± 0.05^a^
35	194.50 ± 0.51^b^	346.00 ± 0.45^b^	19.25 ± 0.03^c^
45	122.33 ± 0.22^c^	374.00 ± 0.53^a^	17.06 ± 0.03^d^
Warming regime			
Warming	263.56 ± 0.16^a^	350.30 ± 0.16^a^	26.57 ± 0.01^a^
Not warming	153.79 ± 0.16^b^	267.76 ± 0.09^b^	19.88 ± 0.01^b^
Sampling time			
Immediately	260.38 ± 0.18^a^	293.79 ± 0.16^b^	22.19 ± 0.01^b^
At the end of storage	156.98 ± 0.14^b^	324.25 ± 0.13^a^	24.26 ± 0.01^a^

Data are means of replicates (*n* = 12 for interruption date in storage and 24 for warming regime and sampling date)  ± standard error. Fruits were stored for 70 days at 2 ± 0.5°C and 90 ± 5% relative humidity. Warming was performed as 1 day at 20°C with 70% relative humidity. Put: putrescine; Spd: spermidine; Spm: spermine. For each experimental factor and evaluated index, means followed by the same letter are not significantly different by the least significant difference (LSD) test, *P* < 0.05.

## Data Availability

All data have been placed in the manuscript.
